# Reassessing the most popularly suggested measurement models and measurement invariance of the Maslach Burnout Inventory – human service survey among Vietnamese healthcare professionals

**DOI:** 10.1080/21642850.2021.2019585

**Published:** 2022-01-05

**Authors:** Thi Hong Thai Bui, Thi Minh Duc Tran, Thi Nhu Trang Nguyen, Thy Cam Vu, Xuan Diep Ngo, Thi Hang Phuong Nguyen, Thi Le Hang Do

**Affiliations:** a Faculty of Psychology, VNU University of Social Sciences and Humanities, Vietnam National University, Ha Noi, Vietnam; b Faculty of Sociology, VNU University of Social Sciences and Humanities, Vietnam National University, Ha Noi, Vietnam; c National Institute of Mental Health, Ha Noi, Vietnam; d Faculty of Psychology, VNU University of Social Sciences and Humanities, Vietnam National University, Ho Chi Minh City, Vietnam; e Faculty of Psychology-Education, University of Science & Education, The University of Da Nang, Da Nang, Vietnam; f VietNam Academy of Social Sciences, Institute of Psychology, Hanoi, Vietnam

**Keywords:** MBI-HSS, burnout, measurement model, measurement invariance, healthcare professionals

## Abstract

**Background:**

Despite its popularity, Maslach Burnout Inventory-Human Service Survey (MBI-HSS)’s factorial structure has been subject to considerable debate, and its measurement invariance (MI) is seldomly examined. This cross-sectional study aims at reassessing the most popularly suggested structures of this instrument, namely the 20- and 22-item three-factor model on Vietnamese healthcare professionals. It also examines the MI of MBI-HSS across genders, occupations, and mental health conditions.

**Method:**

Self-administered questionnaires were sent out to 1500 doctors and nurses working at 15 hospitals in big cities in Vietnam in September and October 2020, and 1162 valid questionnaires were collected. The questionnaire consists of three sets of questions covering (1) demographic information of participants; (2) MBI-HSS questionnaire; and (3) The 21-item version of the Depression-Anxiety-Stress Scale. MBI-HSS scale was validated on Vietnamese sample for the first time; therefore, we used the repeated forward–backward procedure to translate this scale into Vietnamese. To examine which model best fits the data, a series of Confirmatory Factor Analysis (CFA) was used to test the model fit of correlated three-factor model, second-order hierarchical model, and bi-factor model. The reliability of the MBI-HSS was assessed using Cronbach’s *α* coefficients. Then, multiple-group CFA (MGCFA) was applied to determine whether the MBI-HSS has a similar structure between groups different in gender, occupation, and mental health condition.

**Results:**

Our findings confirmed that the 22-item MBI-HSS best fit the data, and this scale measures three distinct but related aspects of burnout, including Emotional Exhaustion, Depersonalization, and Personal Accomplishment. The MI of MBI-HSS across genders and occupations was also confirmed. However, data did not fit well with group at risk for common mental health disorders. It can be concluded that the Vietnamese version of MBI-HSS is a valid measure to assess burnout level of healthcare professionals in Vietnam who are not at risk for mental health disorders.

## Introduction

Burnout is an increasingly alarming issue in modern workplaces, which is related to several common working conditions such as workload and time pressure, role conflict and role ambiguity, lack of social support, or lack of autonomy (Public Health England, 2016). Among occupations, healthcare professionals are especially susceptible to suffer burnout (Bartz & Maloney, 1986; Romani & Ashkar, 2014). Some even affirm that burnout is inevitable in this occupation (Montgomery, 2014). Burnout has been recorded to negatively affect healthcare workers’ mental health and job dissatisfaction (Spence Laschinger & Fida, 2014), reduce their well-being (Schaufeli, Bakker, van der Heijden, & Prins, 2009), consequently has a negative impact on patients’ safety (Panagioti et al., 2018), increase medical errors and medical malpractice suits, and lower interpersonal teamwork (Dyrbye et al., 2018).

Especially in the face of Covid-19 pandemic, burnout becomes an unavoidable challenge for those working in hospital settings globally (Amanullah & Ramesh Shankar, 2020; Gualano et al., 2021). Research documented that the outbreak of Covid-19 pandemic both directly and indirectly correlated to the increase of burnout level among healthcare professionals, by increasing their workload, increasing the fear of being infected, reducing their time for physical activities and relaxation, increasing errors, and hence leading to the increase in litigation which in turn resulted in the increase of occupational stress (Magnavita et al., 2021). This situation thus raises an essential call for establishing some effective intervention strategies to measure, prevent, and reduce burnout for healthcare professionals. Dyrbye et al. (2018) suggest that organizations need to include measures of healthcare professionals’ well-being or burnout to their routine institutional performance measures, such as quality metric, patient satisfaction, or patient volume.

There are quite many instruments to measure burnout. Oldenburg Burnout Inventory, for example, released in 2001 as a response to the Maslach Burnout Inventory (MBI-HSS) for not having negatively worded items, is composed of 16 items constructing two factors as exhaustion and disengagement from work (Demerouti, Nachreiner, & Schaufeli, 2001). Copenhagen Burnout Inventory, released in 2005, has 19 items covering three areas: personal, work, and client-related burnout (Kristensen, Borritz, Villadsen, & Christensen, 2005). More recently, the Stanford Professional Fulfilment Index, released in 2018, measures burnout in specifically physicians (Trockel et al., 2018). Among the existing burnout measures, MBI–Human Services Survey (HSS), released in 1981, is the most widely used in research globally (de Beer et al., 2020). A review by Dyrbye et al. (2018) demonstrated that MBI-HSS has the strongest construct validity data for use for U.S. physicians and other healthcare professionals in comparison with other burnout measures.

MBI-HSS was developed based on the conceptualization of burnout as ‘a syndrome of emotional exhaustion (EE), depersonalization (DP), and reduced personal accomplishment (PA) that can occur among individuals who do ‘people work’ of some kind’ (Maslach & Jackson, 1986, p. 1). The authors of this instrument hold that among the three dimensions of burnout, EE is the core dimension (Maslach & Jackson, 1981). EE refers to workers’ emotion drained because of the demanding interpersonal contacts with other people, whereas DP refers to their cynical and callous attitude toward clients or patients, and lack of PA implies their negative evaluation of their work with clients.

We chose this instrument for three reasons. Firstly, among existing burnout scales, the MBI-HSS focuses on relationship-related factors composing burnout, which is specifically relevant to the situation in Vietnam where healthcare workers experience not only exceptionally intensive work workload (Nguyen Ngoc, Le Thi Thanh, Le Thi, Vu Tuan, & Nguyen Van, 2019) but also great pressure from interpersonal strains such as violent abuse from clients (Pham, 2019) so that doctors have to raise their voice asking for more strict punishment on violent behaviors against doctors and nurses in hospitals. Secondly, this instrument has been so popularly used in studies of burnout that, according to de Beer et al. (2020), it presents in about 90% of empirical papers on this topic. Hence, using this scale can allow further comparative studies of burnout across organizations and societies. Thirdly, in Viet Nam, the burnout scale was only validated on a sample of 430 nurses by Nguyen, Kitaoka, Sukigara, and Thai (2018) with the 16-items MBI–General Survey (MBI-GS), confirming three-factor construct. Even though MBI-GS is the more updated version, it is innovated to measure burnout level of non-human service workers (Schaufeli & Taris, 2005). Therefore, this MBI-GS is not suitable for measuring burnout of human-service professionals such as doctors and nurses who often work with people’s physical illness alongside with psychological issues such as anger or frustration, which in turn makes them drained and exhausted and leads them to burnout (Maslach, Jackson, & Leiter, 1996). Accordingly, we chose MBI-HSS to measure burnout of Vietnamese doctors and nurses.

The objective of this study is to assess psychometric properties and MI of MBI-HSS when being adapted to Vietnamese doctors and nurses. The reason for reassessing its psychometric properties is because several studies using exploratory factor analysis and confirmatory factor analysis (CFA) have validated MBI-HSS in different countries and indicated various constructs. With the 22-item versions, studies found one-factor model (Golembiewski & Munzenrider, 1984); two-factor model (Brookings, Bolton, Brown, & McEvoy, 1985); three-factor model (Beckstead, 2002; Poghosyan, Aiken, & Sloane, 2009); or four-factor structure (Chao, McCallion, & Nickle, 2011; Iwanicki & Schwab, 1981). Meanwhile, some studies suggested discarding some items to increase model’s fit indexes. Therefore, some studies found two-factor model with only 7 items (Kalliath, O'Driscoll, Gillespie, & Bluedorn, 2000), some found a three-factor model with 20 items (Hallberg & Sverke, 2004; Loera, Converso, & Viotti, 2014; Pisanti, Lombardo, Lucidi, Violani, & Lazzari, 2013; Schaufeli & Van Dierendonck, 1993), with 19 items (Gómez García, Alonso Sangregorio, & Lucía Llamazares Sánchez, 2018), with 18 items (Kanste, Miettunen, & Kyngäs, 2006), or with 15 items (Oh & Lee, 2009); while some found a four-factor model with 20 items (Gil-Monte, 2005) and 18 items (Firth, McIntee, McKeown, & Britton, 1985); or five-factor model with 19 items (Densten, 2001). Recently, some authors have affirmed the best fit for bi-factor model which allowed all indicators to load directly on an overall general factor—global burnout, as well as on domain-specific factors such as DP or EE (Mészáros, Adám, Szabó, Szigeti, & Urbán, 2014; Szigeti, Balazs, Bikfalvi, & Urban, 2017; Trigo et al., 2018).

In summary, the instability of this measure suggests that its psychometric properties need to be assessed when we use it on a new population. It is worth to note that among all studies on the psychometric properties of MBI-HSS, two structure models that are most frequently replicated are the three-factor structure with 22 items and, even more popularly, three-factor with 20 items (deleting items 12 and 16). Therefore, we chose to reexamine the psychometric properties of 22- and 20-item versions of MBI-HSS on Vietnamese healthcare professionals in this study.

In order to test if MBI-HSS measures the same construct across all respondents, this study assesses its MI across genders (male vs. female), occupations (doctor vs. nurse), and mental health (being at risk of common health disorders or not). Most of studies assessing MI of MBI-HSS often compare between gender groups. We chose to compare between doctors and nurses since these groups, working in the same organization, differ in many job-related aspects as responsibility, salary, and other benefits. We also compare between groups different in mental health conditions, suggested by recent research of Trigo et al. (2018). This study discovered that depressive disorder could affect the psychometric properties of the MBI-HSS in nursing assistants. The study of Trigo et al. (2018) may be the first study that reported the impact of depression and mental health disorder in general on the psychometric properties of burnout scale. We believe that the association between mental health disorders and healthcare professionals’ perception of their burnout symptoms needs more exploration so that both researchers and practitioners can better understand the way burnout happens in populations different in mental health conditions.

## Method

### Sample size and procedure

There are different rules to determine the adequate sample size to validate a scale. Some authors suggested 10 participants for each item (Nunnally, 1994), or at least 300 respondents after initial pre-testing (Clark & Watson, 1995), or a sample size of 500 is generally considered very good while 1000 and above is excellent for all scales (Comrey & Lee, 2013). As suggested by Comrey and Lee (2013), we targeted to get a sample size of 1000 participants. Given that during the outbreak of Covid-19 pandemic, healthcare professionals experienced an exceptionally high workload and other occupational stressful conditions (Gualano et al., 2021; Magnavita et al., 2021) and hence it is hard to reach out to them; therefore, we applied convenient sampling strategy and sent out 1500 self-reported questionnaires to doctors and nurses working at 15 hospitals in Hanoi, Da Nang, and Ho Chi Minh City, Viet Nam during September and October 2020. Each questionnaire was put in an envelope to ensure confidentiality. Participants were asked to put the questionnaire into the envelope again and sealed it when finishing, then gave it back to our contact person at the hospital. Of 1500 questionnaires sent out, 1162 valid ones were collected (making up a response rate of 77.5%). 65.8% of participants are female.

Repeated forward–backward translation procedure was adopted in this study as advised by Van de Vijver & Hambleton (1996) for burnout scale. The scale was first translated independently into Vietnamese by one organizational psychologist and one clinical psychologist. After that, the two translated MBI-HSS (MP) versions were discussed to create the draft version of MBI-HSS (MP) – Vietnamese. Then, the Vietnamese version was translated back into English by an independent translator who did not know about the tool and compared with the original English version. Next, face validity of the draft MBI-HSS (MP) was assessed among 54 doctors and nurses to test their understanding of the language translation. Participants reported that they understood the items as their intended meaning, thus no further refinement of item content was necessary.

### Measurements and procedures

Participants were invited to answer a questionnaire which consists of three sets of questions:

1. Demographic questions, covering sex, age, marital status, children, job title, and occupations of survey participants.
2. The MBI-HSS for Medical Personal (MP) – MBI-HSS-MP (Maslach et al., 1996). This tool aimed to discover how various healthcare professionals view their job and the patients with whom they work closely. It consists of 22 items with three subscales: EE with 9 items, DP with 5 items and (low sense of) PA with 8 items. Each item is scored using a 7-point Likert scale, from 0 – never, 1 – a few times a year, 2 – once a month, 3 – a few times a month, 4 – once a week, 5 – a few times a week, and 6 – daily.
3. The 21-item version of the Depression-Anxiety-Stress Scale (DASS-21) screens symptoms of depression, anxiety, and stress in community settings (Lovibond & Lovibond, 1995). It comprises three subscales, each with seven items. Items were scored on a 4-point scale ranging from 0 – did not apply to me at all to 3 – applied to me very much, or most of the time. Each subscale score ranged from 0 to 21. The scale was validated among Vietnamese population with a good reliability (Tran, Tran, & Fisher, 2013). In the current study, Cronbach’s *α* coefficients were .89, .84, .86, and .91 for Depression, Anxiety, Stress, and overall scale, respectively.

### Data analysis

**Figure 1.**
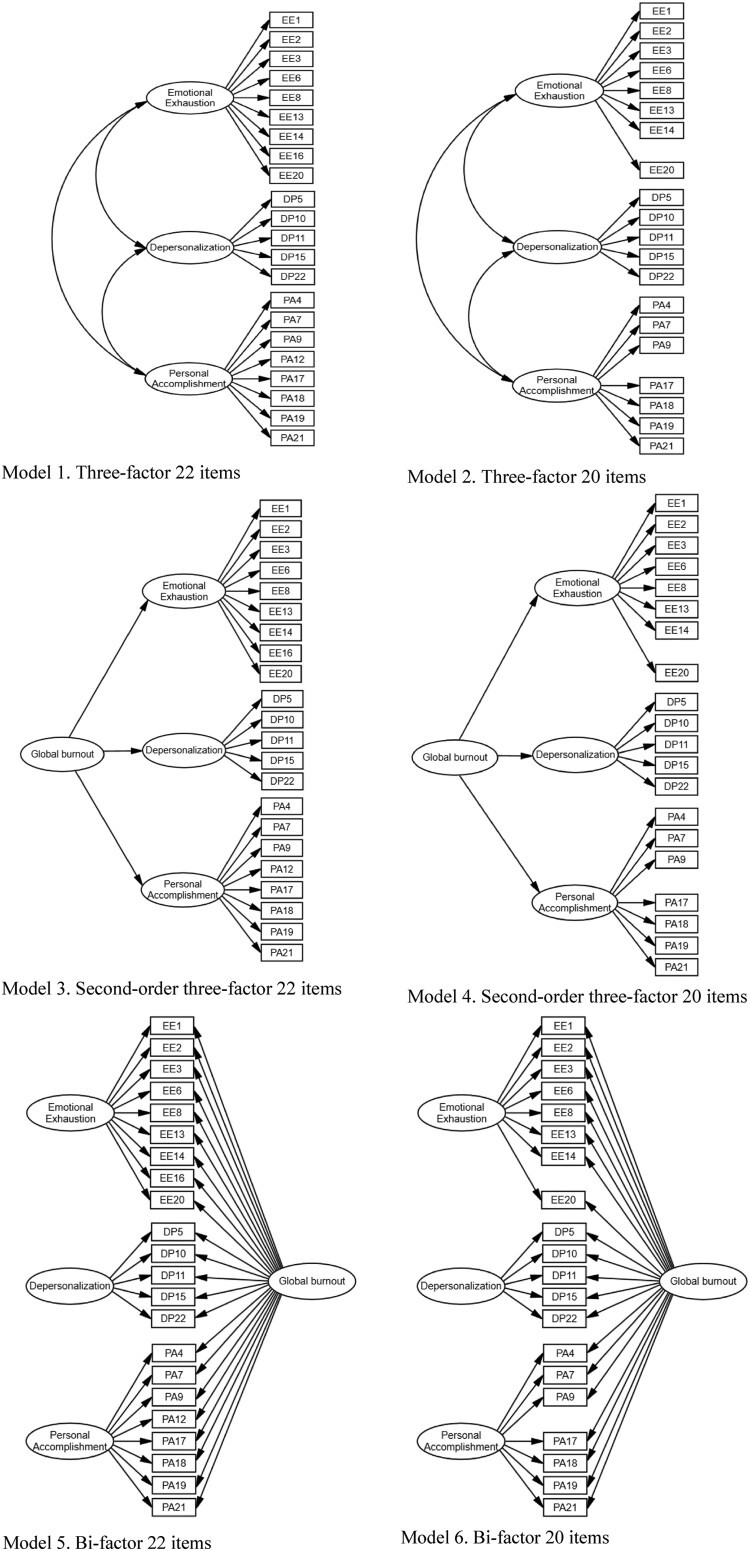
Examination of MBI-HSS different models. Model 1. Three-factor 22 items, Model 2. Three-factor 20 items, Model 3. Second-order three-factor 22 items, Model 4. Second-order three-factor 20 items, Model 5. Bi-factor 22 items, Model 6. Bi-factor 20 items.

We analyzed data, using the 23rd version of the Statistical Package for Social Sciences. The dimensionality of all alternative models of MBI-HSS (MP) was evaluated through CFA with the 23rd version of the SPSS Analysis of Moment Structure (AMOS) software, utilizing the covariance matrix input method of the Maximum Likelihood Estimation (MLE) technique. To identify the model that best fits the data, a series of confirmatory factors analysis were performed. For both version of 22- and 20-item MBI-HSS (minus item 12 and 16), we tested the model fit of correlated three-factor model, of second-order hierarchical model, and of bi-factor model (Figure 1). Several fit indexes were applied to examine satisfactory degree of fit, including Tucker–Lewis index (TLI), comparative fit index (CFI), the root mean square of error approximation (RMSEA), and the standardized root mean square residual (SRMR). We also report *χ*^2^ but do not focus on the significance of the ratio of the Chi-square and its related degree of freedom (*χ*^2^/df), because *χ*^2^ is almost significant, suggesting poor model fit when the sample size is large (Jöreskog, 1993). CFI and TLI values ≥0.90 and 0.95 were considered indicative of acceptable and good model fit, respectively. For the SRMR and RMSEA, value ≤0.10 and 0.08 and ≤0.08 and 0.06, respectively, were considered to reflect acceptable and good fit (Brown & Cudeck, 1993; Hu & Bentler, 1999). Bayesian information criteria (BIC) was also reported, the lower number represents a closer fit.

In order to yield equivalent scores to the full DASS-42, the total score of each scale is multiplied by two and thus ranges from 0 to 42. We applied the cut-off scores suggested by Mental Health Institute – Bach Mai hospital (Hanoi, Viet Nam) and Lovibond and Lovibond (1995) to specify who are at risk for depression (depression score ≥10); anxiety (anxiety score ≥8); and stress (stress score ≥15). Based on participants’ DASS-21 score, we divided them into two groups: one at risk for mental disorder, namely who is at risk for at least one of the above three types of mental health disorder, and one not at risk.

Reliability of the MBI-HSS facets was assessed using Cronbach’s *α* coefficients. Value above 0.70 was considered acceptable for research purposes (DeVellis, 2017).

Finally, we examined the MI of the selected model with multiple-group CFA (MGCFA) to determine whether the MBI-HSS data have a similar structure between groups. In this study, three groups will be examined: male vs. female, doctors vs. nurses, and participants with DASS vs. non-DASS. The MI will be buttressed if the construct of burnout will exhibit no difference between groups. For this analysis, we established the adequacy of the fit indexes of the selected model separately for each sub-group (Byrne, 2012). Next, three levels of MI were examined (Davidov, Meuleman, Cieciuch, Schmidt, & Billiet, 2014): configural invariance indicating that the same factor is measured by the same items across samples, metric invariance showing the meaning of constructs is invariant across samples, and scalar invariance indicating the scale is used in the same mode across samples. The fulfillment of MI means that the selected model is similar across the groups.

### Ethical considerations

The study protocol was reviewed and approved by the Institutional Review Board, Vietnam National University, Hanoi School of Medicine and Pharmacy (approval no. 06/2020/CN-HDDD). All nurses and doctors participated in this study on a volunteer basis and their participation is kept anonymous. All participants received an invitation letter and a leaflet explaining the study and participant’s rights to ensure they fully understood the research and were asked to sign an informed consent form before joining this study.

## Results

### Characteristics of healthcare professionals

65.8% of participants were female. The mean age of participants was 32.12 years with a standard deviation of 8.19 years. 58.5% of participants were married, 39.4% were single, and the others were separated or divorced (2.1%). 36.5% of participants had no child, 19.4% had one child, 32.6% had two children, and 3.3% had three children and over. Two-third of the participants (67.8%) were nurses and the others were doctors. 51.7% worked for the current hospital from 1 to 5 years, 21.0% from 6 to 10 years, 10% from 11 to 15 years, and 17.3% more than 15 years. 11.2% worked at private hospitals, 25.4% at public hospitals, and 63.4% at public hospitals with financial autonomy. Regarding mental health, 31.1% of participants were at risk for at least one of the three mental health issues as screened by DASS-21, namely depression, anxiety disorder, and stress; 68.9% showed no risk for any of the above mental health issues. Table 1.

**Table 1.** Sample characteristics (*N* = 1162).

Variables	Sample
Gender	
Female	762 (65.8%)
Male	400 (34.2%)
Age	21–70 (M = 32.12, SD = 8.19)
Marital status	
Single	458 (39.4%)
Married	680 (58.5%)
Others	24 (2.1%)
Number of children	
0	424 (36.5%)
1	226 (19.4%)
2	379 (32.6%)
3 and over	38 (3.3%)
Missing	95 (8.2%)
Work position	
Nurse	788 (67.8%)
Doctor	374 (32.2%)
Year of experience	
≤5	601 (51.7%)
6–10	244 (21.0%)
11–15	115 (10%)
>15	202 (17.3%)
Type of hospitals	
Private hospitals	130 (11.2%)
Public hospitals without financial autonomy	295 (25.4%)
Public hospitals with financial autonomy	737 (63.4%)
Risk for mental health disorders	
Yes	361 (31.1%)
No	801 (68.9%)

### Confirmatory factors analysis of alternative model of MBI-HSS-MP

Table 2 displays the fit indexes of all the tested models. All 20-item models of MBI-HSS (minus item 12 and item 16) were not supported in the current study. When fitting to the data, the bi-factor 22-item model did not meet acceptable standards (TLI and CFI <0.90). While both three-factor model and second-order hierarchical model demonstrated acceptable fit. The three-factor model (CFI = 0.96, TLI = 0.95, RMSEA = 0.05 [90%CI: 0.05-0.06], and SRMR = 0.06) performed slightly better than the hierarchical three-factor model. In addition, we split the sample into two random sub-samples of equal size (*n* = 518), and CFA results showed that the three-factor model fitted well both data sets (see Figure 3 in Appendix).

**Table 2.** Fit indexes of alternative measurement models of MBI-HSS-MP.

Model	*χ*^2^	df	*p*	CFI	TLI	BIC	RMSEA [90% CI]	SRMR
**Model 1. Three-factor 22 items**	**921**	**167**	**<0.001**	**0**.**96**	**0**.**95**	**1528**	**0.05 [0.05–0.06]**	**0**.**06**
Model 2. Three-factor 20 items	1443	160	<0.001	0.87	0.85	1796	0.08 [0.08–0.09],	0.07
**Model 3. Second-order three-factor 22 items**	**925**	**167**	**<0.001**	**0**.**93**	**0**.**93**	**1650**	**0.07 [0.06–0.07]**	**0**.**06**
Model 4. Second-order three-factor 20 items	1464	161	<0.001	0.86	0.84	1810	0.08 [0.08–0.09]	0.07
Model 5. Bi-factor 22 items	938	167	<0.001	0.89	0.88	1700	0.07 [0.07–0.08]	0.06
Model 6. Bi-factor 20 items	1460	161	<0.001	0.88	0.88	1800	0.08 [0.08–0.09]	0.07

*χ*^2^ – normal theory weighted least squares chi-square; df – degrees of freedom; CFI – comparative fit index; TLI – Tucker–Lewis index; BIC – Bayesian information criteria; RMSEA – root mean square error of approximation; SRMR – standardized root mean square residual.

**Figure 2.**
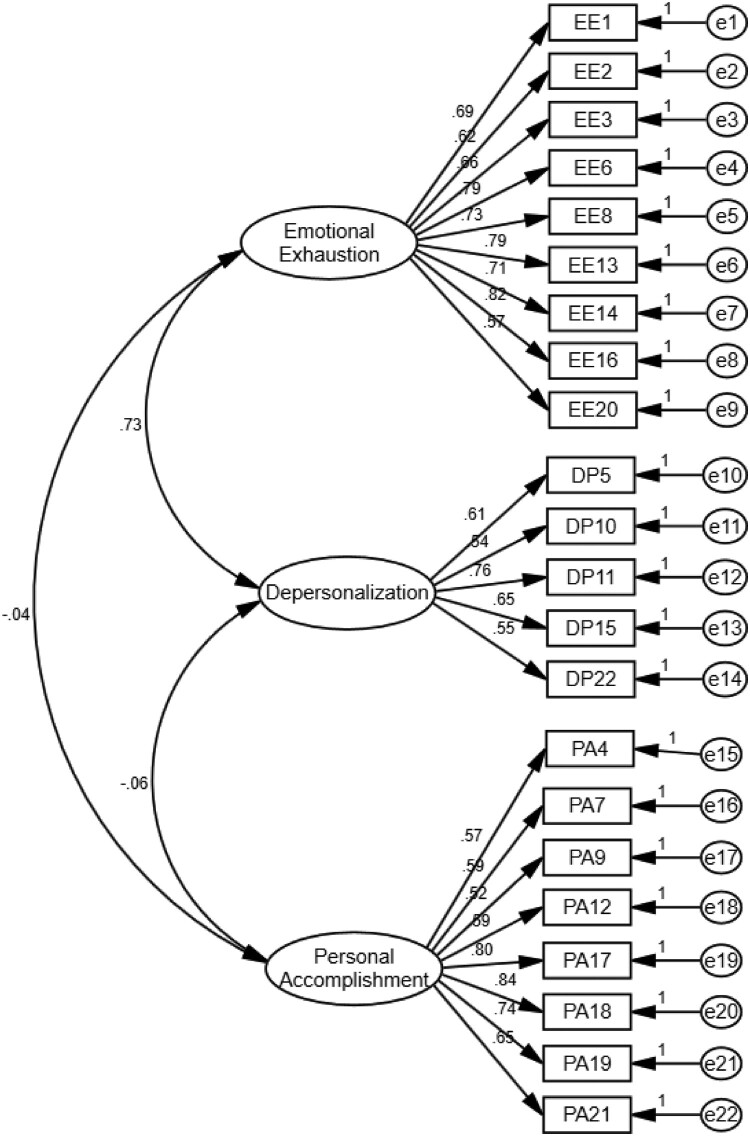
Factor loadings for three-factor model.

Figure 2 displays the standardized factor loadings for the three-factor model. Results showed that all items significantly loaded onto their expected specific dimension with high factor loading. The standardized loadings ranged between 0.57 and 0.82 on EE, between 0.54 and 0.76 on DP, and between 0.52 and 0.84 on PA, showing good quality (Comrey & Lee, 2013). In addition, all dimensions of MBI-HSS demonstrated adequate internal consistency, with Cronbach’s *α* value was 0.91, 0.77, and 0.88 for EE, DP, and PA, respectively. Furthermore, results showed a strong and positive correlation between EE and DP (*r* = 0.725, *p* < 0.001), and a weak and negative correlation between DP and PA (*r* = −0.064, *p* < 0.05), whereas no correlation was found between EE and PA (*r* = −0.038, *p* > 0.05).

### MI of the three-factor model of MBI-HSS

The initial CFA was performed separately for the male and female healthcare professionals and showed acceptable fit indexes for genders (Table 3). Among both men and women participants, the model exhibited an acceptable fit. For men, item loadings ranged from 0.54 to 0.83 on the EE scale, from 0.59 to 0.80 on the Depersonalization scale, and from 0.57 to 0.89 on the PA scale. For women, the item loadings ranged from 0.55 to 0.82, from 0.50 to 0.75, and from 0.50 to 0.82 on the EE, DP, and PA, respectively. Results also show acceptable fit indexes for all the configural, metric, and scalar MI for the three-factor model on both male and female.

**Table 3.** Fit indexes indicating gender, occupations, and status of mental health measurement invariance for the three-factor model of MBI-HSS among Vietnamese healthcare professionals.

Gender group CFA	*χ*^2^	df	*p*	CFI	TLI	RMSEA (90% CI)	SRMR
Men	483	168	<0.001	0.94	0.92	0.07 [0.06–0.08]	0.06
Women	679	168	<0.001	0.94	0.92	0.06 [0.06–0.07]	0.07
Invariance nested model							
Configural (unconstrained model)	1163	336	<0.001	0.94	0.92	0.05 [0.04–0.05]	0.06
Metric (equal factor loadings)	1196	355	<0.001	0.94	0.92	0.05 [0.04–0.05]	0.06
Scalar (equal item intercepts)	1211	361	<0.001	0.94	0.92	0.05 [0.04–0.05]	0.07
Occupational group CFA							
Nurses	718	168	<0.001	0.93	0.91	0.07 [0.06–0.07]	0.07
Doctors	479	168	<0.001	0.94	0.92	0.07 [0.06–0.08]	0.06
Invariance nested model							
Configural (unconstrained model)	1197	336	<0.001	0.94	0.92	0.05 [0.05–0.05]	0.07
Metric (equal factor loadings)	1242	335	<0.001	0.94	0.92	0.05 [0.05–0.05]	0.07
Scalar (equal item intercepts)	1252	361	<0.001	0.94	0.92	0.05 [0.04–0.05]	0.07
Mental health group CFA							
With DASS (*N* = 361)	598	168	<0.001	0.89	0.87	0.09 [0.08–0.09]	0.08
Non-DASS (*N* = 801)	572	168	<0.001	0.95	0.94	0.05 [0.05–0.06]	0.05
Invariance nested model							
Configural (unconstrained model)	1151	334	<0.001	0.94	0.91	0.05 [0.04–0.05]	0.08
Metric (equal factor loadings)	1172	353	<0.001	0.94	0.92	0.05 [0.04–0.05]	0.08
Scalar (equal item intercepts)	1226	359	<0.001	0.93	0.91	0.05 [0.04–0.05]	0.10

Secondly, the CFA was also carried out separately for nurses and doctors. Results indicated acceptable fit indexes for the two groups differentiated by occupations (Table 3). Among both nurses and doctors, the model exhibited an acceptable fit. For nurses, the item loadings on the EE ranged from 0.54 to 0.78, on the DP ranged from 0.53 to 0.75, on the PA from 0.46 to 0.82. For doctors, the item loadings were from 0.50 to 0.85, from 0.55 to 0.80, and from 0.57 to 0.90 on the EE, on the DP, and on the PA, respectively. Results, as presented in Table 3, also demonstrated acceptable fit indexes for all the configural, metric, and scalar MI for the three-factor model for both nurses and doctors.

Remarkably, when fitting the three-factor model on DASS vs. non-DASS participants, almost all indexes do not meet acceptable standards for participants with DASS, while good fit indexes were found for non-DASS group. It means that the three-factor model of MBI-HSS did not fit for healthcare professionals who are at risk for mental health disorders.

## Discussion

Burnout is a psychological response to chronic stress (Maslach, 2004) and is often widespread among healthcare professionals. Therefore, it is necessary to develop a measure to screen burnout for this population. As the first study validating the MBI-HSS (MP) on Vietnamese healthcare professionals, this study examines the construct validity of the instrument with two versions well-established and popularly used in studies on burnout, namely the 22-item 20-item version (discard item 12 and item 16). To test the global fit of the alternative factor structures of the MBI-HSS to the empirical data, we conducted a series of confirmatory factor analyses.

Our results support the original three-factor structure of MBI-HSS with 22 items as provided by Maslach and Jackson (1986). CFA analysis on 1162 Vietnamese doctors and nurses confirmed that the structure—which is composed of EE (9 items), DP (5 items), and PA (8 items)—has an acceptable fit to the data. This finding was in the same line with quite many studies examining MBI-HSS psychometric properties on healthcare professionals (e.g. Beckstead, 2002; Poghosyan et al., 2009). The results also confirmed that the MBI-HSS measures three distinct but related dimensions of burnout. It is noteworthy that existing studies on MBI-HSS suggested different adjustments on the items reserved for each factor and even different structure, however, most of the studies, though conducted in different languages in different countries, confirmed the 3-factor structure (e.g. Hallberg & Sverke, 2004; Kim & Juye, 2008; Loera et al., 2014; Oh & Lee, 2009; Schaufeli, Salanova, González-romá, & Bakker, 2002). Meanwhile, studies proposed one-factor, two-factor, four-factor, or five-factor structures were not replicated.

Reliability assessment using Cronbach’s *α* showed good reliability for EE and acceptable reliability for DP and PA. Previous studies reported the same results (Gómez García et al., 2018; Wheeler, Vassar, Worley, & Barnes, 2011). As reviewed in the introduction, different studies examining psychometric properties of MBI-HSS may come up with different reliability assessments of DP and PA, however, all of them maintain the Exhaustion Dimension (Ferreira Bortoletti et al., 2012). Therefore, some studies only examined the impact of EE on work life of employees such as job satisfaction (Baeriswyl, Krause, & Schwaninger, 2016; Skaalvik, 2020), or job performance (Halbesleben & Bowler, 2007). This finding thus supports the theoretical assumption of MBI-HSS’s authors that EE is the core dimension of burnout (Maslach & Jackson, 1981). However, we argue that burnout should not be measured as a single-dimensional scale. Instead, our results confirmed the multi-dimensionality of MBI-HSS.

In addition, our study documented a strong correlation between EE and DP, mild correlation between DP and PA, and no correlation between EE and PA. This finding provides more evidence for the relative independence of the three dimensions of the construct. In practice, this finding, in the same line with the study of Pisanti et al. (2013), suggests that psychological support for healthcare professionals in dealing with burnout should be strategically specified in each single dimension in order to maximize the effectiveness of intervention.

Another objective of our study was to evaluate the MI of the three-factor model of MBI-HSS through different groups. The results indicated that the three-factor model fitted well for both male and female employees in our sample. This finding was in the same line with the study of Pisanti et al. (2013), In addition, the structure of the three-factor model did not significantly differ on factor loadings and item intercepts between nurses and doctors. To our best knowledge, most of the existing studies examined the psychometric properties of MBI-HSS on only nurse sample, hence the MI of MBI-HSS across occupation groups (e.g. doctors vs. nurses in hospital settings) is hardly evaluated. The findings of this study additionally confirm the stability of three-factor structure on both nurse and doctor groups. Since the confirmation of measure invariance is a prerequisite that should be met before any meaningful comparison can be made between different groups, this finding allows more analyses of the association between burnout and such important factors such as gender and job.

Remarkably, this study found that the data did not fit well with group at risk for common mental health disorders, whereas the data fit well with the group without symptoms of mental health disorder. Even though very few studies examined the measure invariance across group with vs. without risk for mental health disorders, studies such as that of Trigo et al. (2018) also found that MBI-HSS did not fit for depressive persons. These authors argued that depressed mood could affect the subjectivity of the symptom perception and consequently limit the validity of burnout measures. For practical implication, this finding suggests that hospital managers, clinical psychologists, counselors, and hospital social workers, when building up intervention programs to prevent and treat burnout among healthcare workers, need to properly assess their mental health before intervention, and that intervention on reducing burnout should be performed only after providing effective intervention on reducing depression, anxiety, and stress.

In summary, this study contributes to the existing knowledge of burnout among healthcare professionals in that it, when validating the MBI-HSS scale on Vietnamese sample, confirms the three-factor structure with 22 items as originally proposed by the authors of MBI-HSS. More importantly, it provides evidence that this scale does not fit well with individuals with symptoms of mental health issues. This finding suggests a theoretical re-examination of burnout as a state of psychological exhaustion and its correlations with other psychological and mental disorders. This finding also implies that the screening of and intervention with burnout should be conducted after symptoms of common mental health issues are mitigated.

It is worth to note that this study was conducted during the outbreak of Covid-19 pandemic in Vietnam, which means that healthcare professionals were working under exceptionally stressful conditions. Some of them hence experienced some types of mental health issues as depressions, anxiety, and stress as indicated by DASS-21. Therefore, we could observe how these common mental health issues neutralized healthcare professionals’ perception of their burnout state. In a normal condition, it might be more difficult to discover this special association between burnout and the three common mental health issues described in DASS-21. This situation may also explain why so far only few studies documented that burnout scale did not fit well with population with mental health issues as found in the study of Trigo et al. (2018) and this current study.

## Conclusion

Our study confirms that the Vietnamese version of MBI-HSS (MP) measures three distinct but related aspects of burnout, including EE, DP, and PA, and replicate the original 22-item three-factor structure of MBI-HSS (MP). Since the psychometric properties of the Vietnamese version of MBI-HSS was satisfactory, this study suggests that it can be used by not only researchers interested in studying issues related to burnout but also organizational psychologists as a valid and reliable measure to assess burnout among Vietnamese healthcare professionals. Furthermore, these results provide some practical suggestions for hospital managers, asking them to recognize not only burnout level of their employees but also the three different and related aspects of burnout in order to offer effective intervention strategies to prevent and reduce burnout among healthcare workers, especially in the context where burnout is becoming one of greatest challenges for doctors and nurses as in Vietnam.

Last but not least, this study confirms the psychometric equivalence of MBI-HSS (MP) across genders and occupations. However, it finds that mental health disorders may affect the way healthcare professionals perceive their burnout symptoms, hence the application of MBI-HSS (MP) on high-risk group should be conducted with caution. It is recommended that practitioners working with high-risk group should treat their mental health disorder before using MBI-HSS (MP) to assess and treat their burnout symptoms.

## Limitations and recommendations for future research

Although the sample of this study was quite large and collected from various workplaces in three regions of Vietnam, is only representative for healthcare professionals working in big cities where workplace conditions as well as pressure may very different from small cities and rural areas. We recommend that future research may try to apply the 22-item Vietnamese version of MBI-HSS on both healthcare professionals and other workers in the field of human services in more various contexts (e.g. rural vs. urban, big cities vs. small cities). Secondly, of all participants, we were able to contact 392 nurses and doctors who voluntarily left us their personal contact, and among these participants, only 64 were willing to fill in the questionnaire for the second time. Therefore, we could not conduct the test–retest reliability of MBI-HSS. Future studies can examine this index. Thirdly, there is no study validating another burnout scale in Vietnam so far, hence it is unable for us to check the concurrent validity of MBI-HSS-MP. However, we hope that this study is a beginning step promoting the validation of burnout scales in Vietnam, and thus providing assessment tools for those working with or studying burnout in Vietnam. Finally, since the study was conducted on a large sample and hence participants’ mental health was self-reported using DASS-21 scale instead of being individually diagnosed by psychiatrists to make sure if they had mental health disorders with clinical symptoms described in DSM-V or ICD-10. This way of screening mental health may affect the reliability of categorizing participants into group at risk for common mental health disorder.

Despite the above limitations, this study has made some significant contributions. Besides confirming three-factor structure of MBI-HSS in assessing burnout level of healthcare professionals, this study has provided more evidence on the impact of common mental health disorders on Maslach burnout scale’s psychometric properties and confirmed the measure invariance of this scale across genders and occupations, which allows meaningful comparison of burnout level across different groups.

## Figures and Tables

**Figure A1.**
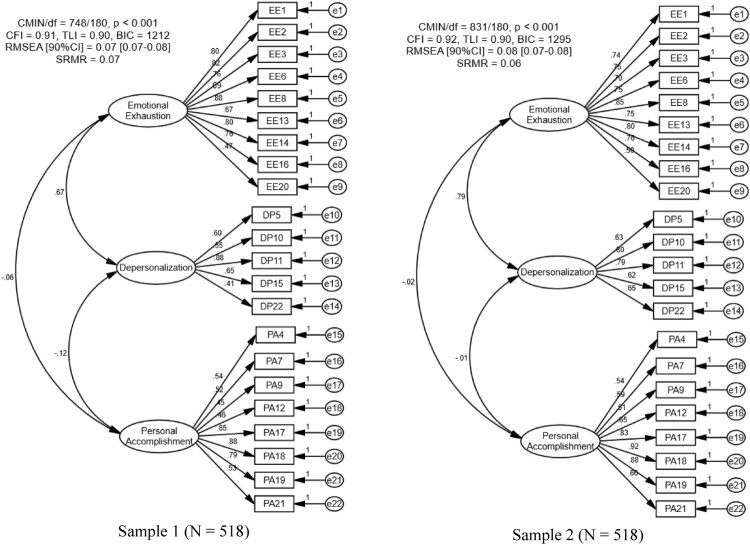
Fit index and factor loadings for three-factor model on two random sub-samples.

## Appendix

Results of Fit index and factor loadings for three-factor model on two random sub-samples.
